# Inter- and intra-site reliability of forearm sweating induced by pilocarpine iontophoresis

**DOI:** 10.1016/j.jphyss.2026.100089

**Published:** 2026-07-23

**Authors:** Hui Wang, Lu Meng, Junto Otsuka, Hanano Kato, Tatsuro Amano

**Affiliations:** a Laboratory for Exercise and Environmental Physiology, Faculty of Education, Niigata University, Niigata, Japan; b School of Human and Social Sciences, Fukuoka Prefectural University, Fukuoka, Japan

**Keywords:** Sweat rate, Measurement error, Reliability

## Abstract

We examined the inter- and intra-site reliability of pilocarpine-induced forearm sweat rate at three concentrations. Under thermoneutral conditions, 10 males and 4 females received 0.001%, 0.01%, and 1% pilocarpine via iontophoresis at the lateral and medial left forearm and the medial right forearm. Sweat rate was measured for 30 min. Inter-site (right vs. left medial) and intra-site (left lateral vs. medial) comparisons were assessed using intraclass correlation (ICC) and coefficient of variation (CV). Inter-site comparisons showed significant correlations at 0.01% and 1% (both *p* < 0.001, *r* ≥ 0.688) with good agreement (ICC ≥ 0.79, CV ≤ 21.5%), whereas 0.001% showed poorer reliability (CV = 47.3%). Intra-site reliability was highest at 1% (ICC = 0.89, CV = 16.5%) and lower at 0.01% and 0.001%. These findings indicate that inter-site comparisons of pilocarpine-induced sweating at 1% and 0.01% concentrations provide reliable responses for sweat mechanism studies (e.g., with and without antagonist administration).

## Introduction

Local sweat rate is widely measured to evaluate sudomotor function and thermoregulatory responses under various physiological and pharmacological conditions [1], [2], [3]. Quantifying local sweating induced by sudorific agents enables investigation of the mechanisms regulating sweat gland function [4], [5], [6]. In particular, comparing responses between a pharmacologically treated site and an untreated control site can provide insight into the local mechanisms of sweating. To deliver such agents, previous studies have commonly used iontophoresis [7], [8] or intradermal microdialysis [5], often at the forearm. These approaches generally assume that local sweat responses are sufficiently similar between right and left forearms and between nearby forearm sites. Indeed, Kenefick et al. [9] observed comparable inter-site forearm sweating during steady-state walking in the heat following acclimation, implying inter-site similarity when thermoregulatory drive is elevated. However, such whole-body exercise elicits sweating through central sympathetic activation, and it may not translate directly to local pharmacological stimulation [10].

In contrast, a study indicates that regional variation of sweating may exist within the forearm. Kimura et al., [11] reported that the sweating responses to a high dose of acetylcholine (≥ 100 mM) delivered via microdialysis were greater at distal than proximal forearm regions, whereas the responses to methacholine were attenuated distally. Such variability may reflect regional differences in sweat gland density, cholinergic sensitivity, or neural innervation, although the mechanisms remain unclear. These findings raise concern that the within-forearm site (e.g., distal vs. proximal sites) may not always yield equivalent pharmacological responses. Moreover, the level of cholinergic stimulation produced by varying concentrations of muscarinic agonists may alter sweat output and potentially influence regional variations [11]. Whether the intra- and inter-site reliability of forearm sweating varies according to agonist concentration has not been determined.

The present study evaluated the inter-site (right vs. left forearm) and intra-site (medial vs. lateral forearm) reliability of cholinergic sweating induced by pilocarpine iontophoresis at low, moderate, and high concentrations. Based on previous findings showing similar inter-site forearm sweating responses during exercise [9] and findings suggesting smaller within-forearm differences at low-to-moderate concentrations of muscarinic agonists [11], we hypothesized that sweat rate would show acceptable agreement for both inter- and intra-site comparisons, with greater agreement at low and moderate pilocarpine concentrations for intra-site comparisons.

## Methods

### Ethical approval

The present study was approved by the human ethical committee of Niigata University (reference#: 2018–0069) and conducted according to the latest version of the Declaration of Helsinki. Verbal and written informed consent were obtained from all participants before they participated in the experimental sessions.

### Participants

Ten males and four females participated in this study (age, 25 ± 5 years; height, 169.2 ± 8.4 cm; and weight, 60.9 ± 8.1 kg). All participants were free from any known cardiovascular, metabolic, or neurologic diseases and were non-smokers. To minimize potential seasonal effects on sudomotor function, all experiments were conducted within the same winter season in Niigata, Japan. Because there were no directly comparable previous studies from which to estimate an appropriate expected intraclass correlation coefficient (ICC) for the present design, a formal a priori ICC-based sample size calculation was not performed. Sample size was therefore determined pragmatically based on feasibility.

### Materials and reagents

0.001%, 0.01%, and 1% pilocarpine (muscarinic receptor agonist, Tokyo Chemical Industry, Tokyo, Japan) solution was prepared in Japanese Pharmacopoeia grade distilled water (Otsuka distilled water; Otsuka Pharmaceutical, Tokyo, Japan). The pilocarpine solution was sterilized using a syringe filter with a 0.45 µm membrane (Advantec, Tokyo, Japan). Then the pilocarpine was applied to the skin using a plastic capsule (3.14 cm^2^) containing 1.5 mL of pilocarpine solution absorbed into cellulose cloth (Scotch-Brite; 3 M, Saint Paul, USA). Pilocarpine was administered to the skin via anodal iontophoresis (5 min, 1.0 mA).

### Experimental protocol

Participants were instructed to abstain from alcohol and caffeine consumption and to avoid strenuous physical activity for at least 24 h prior to experimental trials. They were instructed to consume ≥ 300 mL of water the night before and roughly 2 h before arriving at the laboratory. Upon arrival, urine samples were collected to assess hydration status, with euhydration confirmed by a urine-specific gravity of ≤ 1.025 [12]. They then measured height and body weight.

**Fig. 1:**
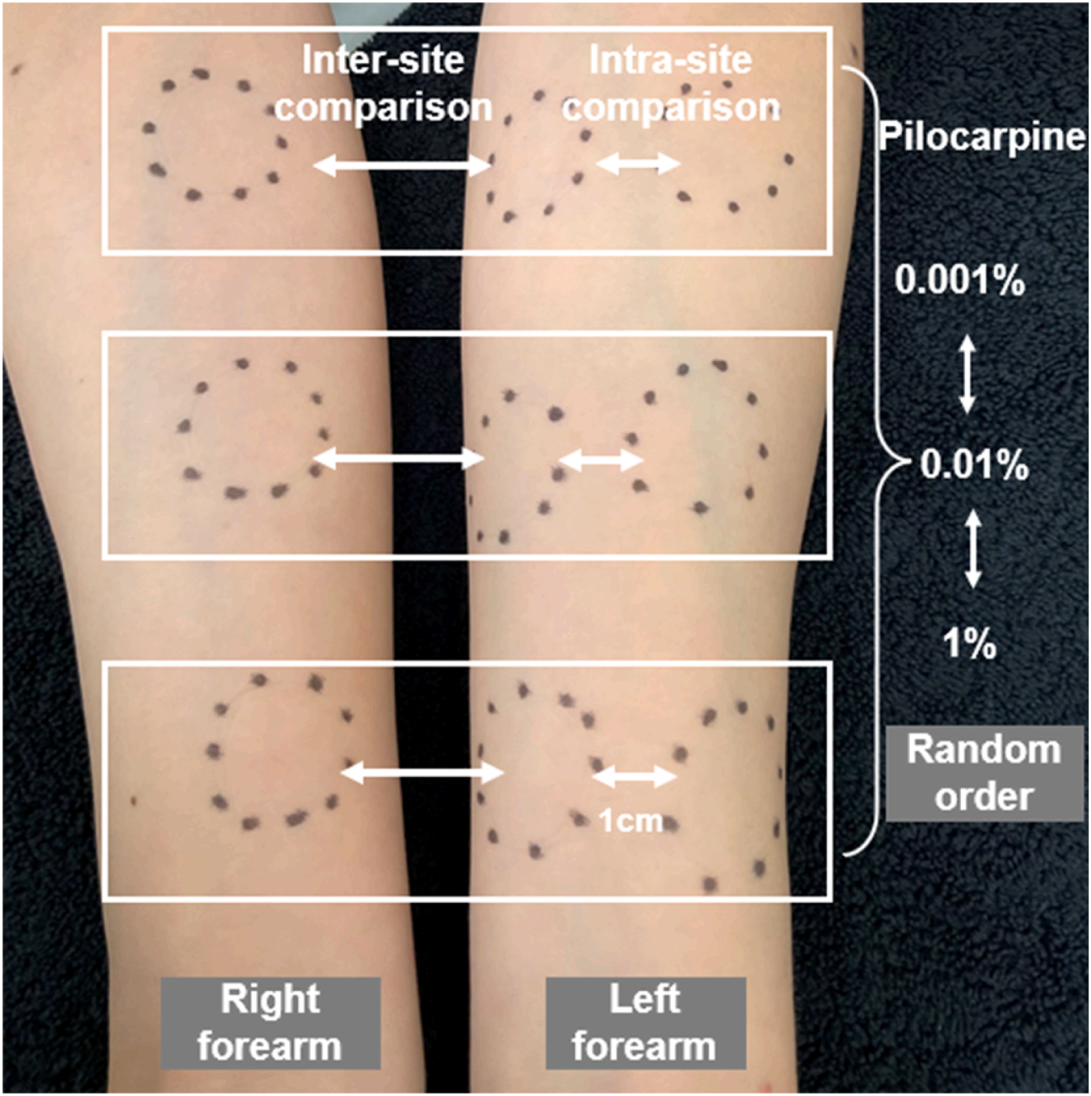
Schematic illustration of the pilocarpine administration protocol. For the inter-site comparison, 0.001%, 0.01%, or 1% pilocarpine was applied to the medial sites of the right and left forearms. The position of the medial site (distal, middle, or proximal forearm) was randomized across trials, but the same relative position was used on the right and left forearms for each concentration. For the intra-site comparison, the same concentration used at the left medial site was also applied to the lateral site of the left forearm at the same relative forearm level.

Participants were seated in a climate chamber regulated at 25°C and relative humidity of 40% for at least 25 min, during which the necessary equipment was prepared. They then received 0.001%, 0.01%, and 1% pilocarpine via iontophoresis at the lateral and medial sites of the left forearm and the medial site of the right forearm, in randomized order (Fig. 1). Specifically, for the inter-site comparison, 0.001%, 0.01%, or 1% pilocarpine was applied to the medial sites of the right and left forearms. The position of the medial site (distal, middle, or proximal forearm) was randomized across trials, but the same relative position was used on the right and left forearms for each concentration. For the intra-site comparison, the same concentration used at the left medial site was also applied to the lateral site of the left forearm at the same relative forearm level. The lateral site was selected on a hair-sparse area while avoiding visible hair as much as possible. The medial and lateral sites were separated by approximately 1 cm, whereas the distal, middle, and proximal forearm positions were separated by approximately 2–3 cm along the longitudinal axis of the forearm. Following each pilocarpine application, a sweat capsule was attached to the pilocarpine-treated sites to measure sweat rate for 30 min. This duration was selected to capture the main pilocarpine-induced sweating response, including the peak and early post-peak phase. The 30-min averaged response was used as a stable measure of local sweat responsiveness.

### Measurements

The height and body weight were measured using a stadiometer (YS501-P; Sanyu, Tokyo, Japan) and scale (HW−100KC; A & D, Tokyo, Japan), respectively. Local sweat rate was measured continuously using the ventilated capsule technique. A 3.14 cm^2^ plastic capsule was affixed at each skin site using a velcro elastic strap. Dry nitrogen gas was passed through each capsule over the skin surface at a rate of 1.2 L·min^−1^. The water content from the effluent air was measured using a capacitance hygrometer (HMT333; Vaisala, Helsinki, Finland). Local sweat rate was calculated using the water content of the effluent air multiplied by the flow rate and normalized for the skin surface area under the capsule (in mg·min^−1^·cm^−2^). Sweat rate data were recorded at 1-s intervals using a data logger system (MX100; Yokogawa, Tokyo, Japan).

### Data and statistical analyses

Reliability between forearm locations was assessed using the ICC calculated based on a two-way random model for single measurements with absolute agreement [ICC(2,1)] [13]. ICC values ≥ 0.70 were considered acceptable for research purposes [14]. The other reliability measure included the coefficient of variation (CV), which represents the magnitude of variation in measurements across sites. CV was calculated as the ratio of the standard deviation to the mean and expressed as a percentage. CV values ≤ 10%, 10–25%, and ≥ 25% were interpreted as indicating good, moderate, and poor reliability, respectively [15].

The minimum detectable change (MDC) was also calculated as previously described by Beaton [16]. The MDC reflects the minimum change that exceeds measurement error. The MDC can be calculated for interpreting differences in individual scores (Eq. 1) or differences in group means (Eq. 2).

(1) $${\mathrm{MDC}}_{\mathrm{indiv}\;}=\;\sqrt{2}\;\times z\;(1-\frac{\;\alpha }{2}\;)\;\times \mathrm{SEM}$$

(2) $${\mathrm{MDC}}_{\mathrm{group}\;}=\;\sqrt{2}\times z\;(\;1-\frac{\;\alpha }{2})\;\times \;\frac{\mathrm{SEM}}{\sqrt{n}}$$

$$\mathrm{SEM}\;=\;\mathrm{SD}\times \;\sqrt{1-\mathrm{ICC}}$$

where the √2 denotes the uncertainty due to two change scores, z(1 − α/2) indicates the z score for a given confidence level (1.96 for 95% confidence interval), SEM represents the standard error of the measurement, and√n refers to the square root of the sample size in the group. We provide both MDC calculations; MDC_indiv_ are provided in the body of the article. Meanwhile, MDC_group_ and Bland-Altman plots are presented in the Supporting information (https://figshare.com/s/10c0f19d26535f4f8b20) and supplementary file.

The sweat rate was calculated as the average of 30-min measurement period. A normal distribution was confirmed based on the Q-Q plot assessment. A paired *t*-test was performed to compare the sweat rate within inter- (i.e., right vs. left medial sites) and intra- (i.e., left lateral vs. medial sites) forearms. To assess the similarity of responses between measurement sites, we examined the linear association between sites using Pearson’s product-moment correlation coefficient. Data were presented as means ± SD, and statistical significance was set at *P* < 0.05. Statistical analyses were performed using SPSS (version 24.0; IBM Corp., Armonk, NY, USA) and Prism (version 10; GraphPad Software, San Diego, CA, USA).

## Results

### Inter-site comparisons (right vs. left medial forearms)

**Fig. 2:**
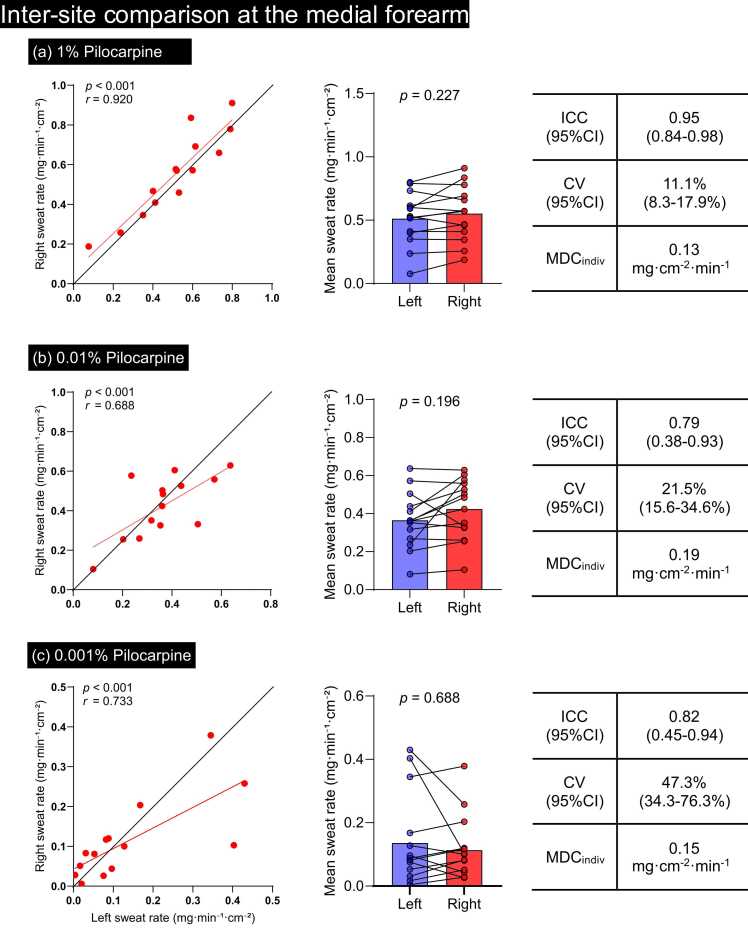
The correlation, mean sweat rate, and reliability metrics of local sweat rate between the left and right medial forearms under three pilocarpine concentrations (n = 14). ICC, intraclass correlation coefficient. CV, coefficient of variation. MDC_indiv_, minimum detectable change at individual levels. Values are presented as mean ± SD.

Local sweat rate demonstrated the strongest positive association between the left and right medial forearms under 1% pilocarpine stimulation (*r* = 0.920, *p* < 0.001; Fig. 2a), followed by 0.001% and 0.01% pilocarpine (*r* = 0.733 and 0.688, respectively; both *p* < 0.001; Fig. 2b, c). Mean sweat rate did not differ between sites at any pilocarpine concentration (all *p* ≥ 0.196; Fig. 2a-c). Inter-site ICCs were excellent at all pilocarpine concentrations (all ICC ≥ 0.75). Inter-site CV was moderate at 1% and 0.01% concentrations, but poor at 0.001% pilocarpine (Fig. 2). Inter-site MDC_indiv_ was 0.13, 0.19, and 0.15 mg·cm^⁻2^·min^⁻1^ for 1%, 0.01%, and 0.001% pilocarpine concentrations, respectively (Fig. 2). Bland–Altman analysis also demonstrated minimal systematic bias between inter-site forearm measurements across all pilocarpine concentrations (mean bias ranging from −0.06 to 0.02 mg·cm⁻^2^·min⁻^1^; Supplementary file: Fig. S1 a-c). Most individuals fell within the limits of agreement for each comparison.

### Intra-site comparisons (left lateral vs. medial forearm)

**Fig. 3:**
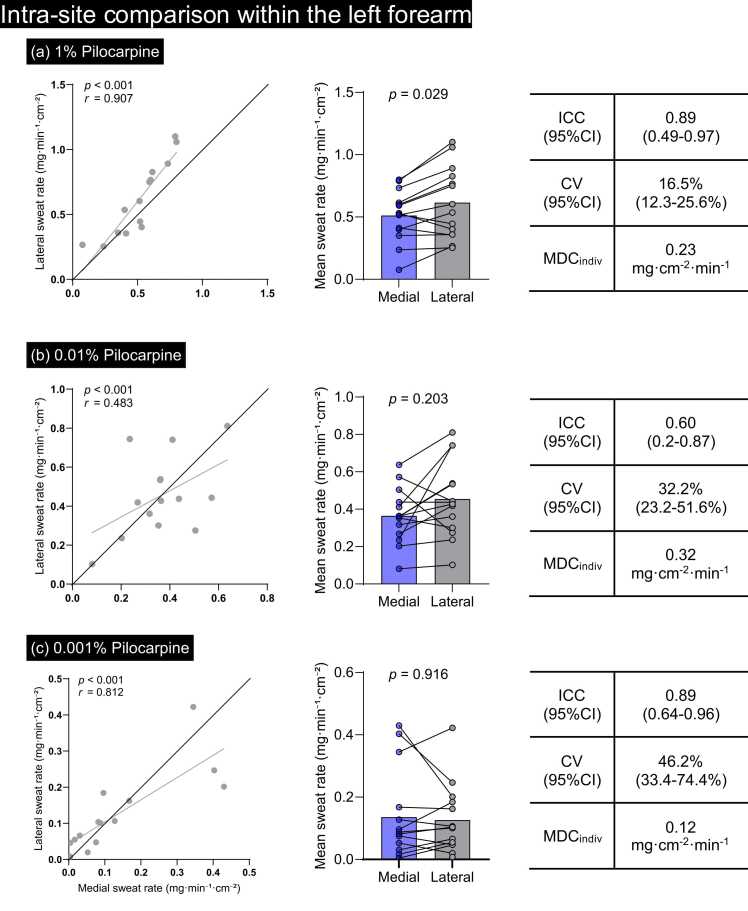
The correlation, mean sweat rate, and reliability metrics of local sweat rate between the lateral and medial left forearm under three pilocarpine concentrations (n = 14). ICC, intraclass correlation coefficient. CV, coefficient of variation. MDC_indiv_, minimum detectable change at individual levels. Values are presented as mean ± SD.

Local sweat rate showed a strong positive association between the medial and lateral left forearms under 1% pilocarpine stimulation (*r* = 0.907, *p* < 0.001; Fig. 3a), followed by 0.001% and 0.01% pilocarpine (*r* = 0.812 and 0.483, respectively; both *p* < 0.001; Fig. 3b, c). Mean sweat rate was marginally higher at the lateral than medial forearm site under the 1% pilocarpine condition (0.55 vs. 0.51 mg·cm^−2^·min^−1^, *p* = 0.029, Fig. 3a), whereas no site difference was observed at the 0.01% and 0.001% pilocarpine concentrations (*p* = 0.203 and 0.916, respectively; Fig. 3b, c). Intra-site ICC was excellent under 1% and 0.001% pilocarpine stimulation (both ICC ≥ 0.75), but only moderate at the 0.01% concentration (Fig. 3). Intra-site CV was acceptable only at the 1% pilocarpine concentration, whereas CVs at 0.01% and 0.001% indicated poor reliability (Fig. 3). Intra-site MDC_indiv_ was 0.23, 0.32, and 0.12 mg·cm^⁻2^·min^⁻1^ for the 1%, 0.01%, and 0.001% pilocarpine concentrations, respectively (Fig. 3). Furthermore, comparisons within the intra-site forearm also showed small mean biases (−0.10–0.01 mg·cm⁻^2^·min⁻^1^, Supplementary file: Fig. S1 d-f) across all pilocarpine concentrations. Most individuals fell within the limits of agreement for each comparison.

## Discussion

We demonstrated that inter-site forearm comparisons showed acceptable reliability in response to 1% and 0.01% pilocarpine, as indicated by high ICC values (ICC = 0.95 and 0.79) together with relatively low CV (CV = 11.1 and 21.5%). In contrast, inter-site reliability was reduced at the 0.001% concentration (CV = 47.3%). For intra-site comparisons, 1% pilocarpine showed acceptable reliability between the medial and lateral sites, whereas reliability was reduced at the lower concentrations (0.01% and 0.001%). These findings provide practical guidance for the design and interpretation of pharmacological studies of cholinergic sweating that compare forearm sites, such as control and antagonist-treated conditions.

Most pharmacological studies of human sweating administer agents into the skin using iontophoresis [6], [17], intradermal microdialysis [18], [19], or intradermal injection [20], and typically compare responses across different skin sites between forearms [9] or within the forearm [11]. One exception used repeated intradermal electrical stimulation combined with microdialysis, which enabled repeated evaluations within the same skin region under control and antagonist conditions [21]. In contrast, repeated iontophoretic administration at the same skin site may not be ideal. In our preliminary observations, sweat rate appeared to be reduced when acetylcholine iontophoresis was repeated at the same forearm site after a 1-h interval, possibly due to desensitization of muscarinic receptors following the initial stimulation. Although this was not formally evaluated in the present study, this practical limitation motivated our interest in examining inter-site reliability of cholinergic sweating within and between forearm regions.

From a methodological perspective, our results suggest that 1% and 0.01% pilocarpine are preferable when comparing sweating responses between the right and left forearms. Under these conditions, site differences exceeding 0.13 and 0.19 mg·cm⁻^2^·min⁻^1^, respectively, may be interpreted as meaningful at the individual level. For example, muscarinic antagonism with atropine is expected to nearly abolish pilocarpine-induced sweating [22], which would clearly exceed the MDC values observed in the present study. In contrast, if a pharmacological intervention induces only a small reduction in sweat rate (e.g., smaller than 0.13 mg·cm⁻^2^·min⁻^1^ under the 1% pilocarpine condition), the current iontophoretic approach may not be sufficiently sensitive to detect that change beyond measurement error. Thus, the present reliability data are most practically useful when the expected pharmacological effect exceeds the corresponding MDC. In addition, these MDC values may also help inform sample size estimation in future pharmacological studies of sweating.

In contrast, 0.001% pilocarpine showed less robust inter-site reliability. One possible explanation for this concentration-dependent response is that higher concentrations of muscarinic agonists would recruit a greater number of sweat glands and thereby produce a more stable and consistent sweating response across sites [23]. In isolated human eccrine sweat glands, some glands respond poorly to low-to-moderate doses of methacholine, whereas nearly all glands are activated at higher doses [23]. Thus, the greater reliability observed at higher pilocarpine concentrations in the present study may reflect more complete sweat gland recruitment. Notably, two individuals with the highest sweat rates at the 0.001% pilocarpine concentration (Fig. 2) showed substantially deviant inter-site responses. However, a post hoc sensitivity analysis excluding these two individuals increased the ICC and reduced the CV, but the CV remained > 25%. This indicates that they did not fully explain the poor absolute reliability observed at this concentration.

Moreover, the discrepancy between a relatively high ICC (2,1) value (0.82) and an elevated CV (47.3%) at the 0.001% concentration likely reflects the small mean sweat response. Although ICC (2,1) assesses absolute agreement, it is also influenced by between-subject variability, whereas CV reflects variability relative to the mean response. Thus, despite acceptable absolute agreement by ICC, the high CV indicates poor practical precision at this low concentration.

For intra-site forearm comparisons between the medial and lateral forearms, 1% pilocarpine appeared to provide the most reliable response. This may likewise reflect greater sweat gland recruitment at higher levels of muscarinic stimulation. However, this differs somewhat from the findings of Kimura et al. [11], who reported that within-forearm differences between distal and proximal sites emerged only at higher agonist doses and were absent at lower doses. Differences in site orientation (distal-proximal vs. medial-lateral), drug delivery method (microdialysis vs. iontophoresis), or agonist type (acetylcholine, methacholine, or pilocarpine) may contribute to these discrepancies. Therefore, the optimal methodological conditions for local pharmacological sweating studies may depend on the specific experimental question and setup. Finally, there are some limitations to the present study that should be noted. First, we did not perform a formal a priori ICC-based sample size calculation. This may have contributed to relatively wide confidence intervals for some reliability estimates; therefore, these findings should be interpreted with caution. Second, because only four female participants were included, the present study could not evaluate potential sex differences in site-to-site reliability.

In summary, inter-site forearm comparisons using pilocarpine iontophoresis at 1% and 0.01% appear to be suitable for studies examining local mechanisms of sweating. By contrast, lower pilocarpine concentrations and intra-site forearm comparisons between medial and lateral sites require greater caution because of their lower reliability. These considerations may help researchers optimize site selection, estimate sample size, and interpret pharmacological sweating responses more appropriately.

## Fundings

This study was supported by a grant from Grant-in-Aid for Scientific Research (nos. 24K02828 and 21H03317) from the Japan Society for the Promotion of Science from the Ministry of Education, Culture, Sports, Science, and Technology of Japan.

## Declaration of Competing Interest

No conflicts of interest, financial or otherwise, are declared by the authors.

## Data Availability

Source data for this study are openly available at https://figshare.com/s/10c0f19d26535f4f8b20.

## Footnotes

Appendix A Supplementary data associated with this article can be found in the online version at doi:10.1016/j.jphyss.2026.100089.
