# Supplementary material for: Inter- and intra-site reliability of forearm sweating induced by pilocarpine iontophoresis
Source: J Physiol Sci. 2026 Jul 23;76(3):100089. doi: 10.1016/j.jphyss.2026.100089 (PMC13446331; doi:10.1016/j.jphyss.2026.100089)
Supplement: Supplementary file 1 [file mmc1.docx]

**Figure S1:** Bland–Altman plots showing agreement of sweat rates between inter-site of the forearm (Fig. a- c) and intra-site of the forearm (Fig. d- f) following iontophoresis with 1%, 0.01%, and 0.001% pilocarpine. Solid horizontal lines indicate the mean bias, and dashed lines represent the 95% limits of agreement (mean bias ± 1.96 SD).
